# Transcriptome analysis of orange-spotted grouper (*Epinephelus coioides*) spleen in response to Singapore grouper iridovirus

**DOI:** 10.1186/1471-2164-12-556

**Published:** 2011-11-12

**Authors:** Youhua Huang, Xiaohong Huang, Yang Yan, Jia Cai, Zhengliang Ouyang, Huachun Cui, Peiran Wang, Qiwei Qin

**Affiliations:** 1 Key Laboratory of Marine Bio-resources Sustainable Utilization, South China Sea Institute of Oceanology, Chinese Academy of Sciences, 164 West Xingang Road, Guangzhou 510301, PR China; 2 State Key Laboratory of Biocontrol, School of Life Sciences, Sun Yat-sen University, 135 West Xingang Road, Guangzhou 510275, PR China

## Abstract

**Background:**

Orange-spotted grouper (*Epinephelus coioides*) is an economically important marine fish cultured in China and Southeast Asian countries. The emergence of infectious viral diseases, including iridovirus and betanodavirus, have severely affected food products based on this species, causing heavy economic losses. Limited available information on the genomics of *E. coioides *has hampered the understanding of the molecular mechanisms that underlie host-virus interactions. In this study, we used a 454 pyrosequencing method to investigate differentially-expressed genes in the spleen of the *E. coioides *infected with Singapore grouper iridovirus (SGIV).

**Results:**

Using 454 pyrosequencing, we obtained abundant high-quality ESTs from two spleen-complementary DNA libraries which were constructed from SGIV-infected (V) and PBS-injected fish (used as a control: C). A total of 407,027 and 421,141 ESTs were produced in control and SGIV infected libraries, respectively. Among the assembled ESTs, 9,616 (C) and 10,426 (V) ESTs were successfully matched against known genes in the NCBI non-redundant (nr) database with a cut-off E-value above 10^-5^. Gene ontology (GO) analysis indicated that "cell part", "cellular process" and "binding" represented the largest category. Among the 25 clusters of orthologous group (COG) categories, the cluster for "translation, ribosomal structure and biogenesis" represented the largest group in the control (185 ESTs) and infected (172 ESTs) libraries. Further KEGG analysis revealed that pathways, including cellular metabolism and intracellular immune signaling, existed in the control and infected libraries. Comparative expression analysis indicated that certain genes associated with mitogen-activated protein kinase (MAPK), chemokine, toll-like receptor and RIG-I signaling pathway were alternated in response to SGIV infection. Moreover, changes in the pattern of gene expression were validated by qRT-PCR, including cytokines, cytokine receptors, and transcription factors, apoptosis-associated genes, and interferon related genes.

**Conclusion:**

This study provided abundant ESTs that could contribute greatly to disclosing novel genes in marine fish. Furthermore, the alterations of predicted gene expression patterns reflected possible responses of these fish to the virus infection. Taken together, our data not only provided new information for identification of novel genes from marine vertebrates, but also shed new light on the understanding of defense mechanisms of marine fish to viral pathogens.

## Background

The orange-spotted grouper (*E. coioides*), an important cultured marine fish with a high market value, is an ideal model for studying sex differentiation and reproduction [1,2]. Rapid expansion of aquaculture has, however, led to an increased incidence of disease outbreaks in recent years [3,4]. Emerging viral infectious diseases, including iridovirus and nodavirus, have caused serious damage to the grouper aquaculture industry with mortality rates due to iridovirus infections ranging from 30% (adult fish) to 100% (fry) [5-7]. To date, three iridoviruses that were isolated from diseased groupers have been characterized: Singapore grouper iridovirus (SGIV), orange-spotted grouper iridovirus (OSGIV) and Taiwan grouper iridovirus (TGIV) [5,6,8]. Nevertheless, the molecular mechanisms associated with iridovirus pathogenesis and virus-host interactions are largely unknown, due to the limited amount of available genomic information on *E. coioides*.

Rapid progress in next-generation sequencing technologies can be used for large-scale efficient and economical production of ESTs. *De novo *transcriptome sequencing using 454 pyrosequencing has thus become an important method for studying non-model organisms [9-12]. Transcriptome sequencing facilitates functional genomic studies, including global gene expression, novel gene discovery, assembly of full-length genes, and single nucleotide polymorphism (SNP) discovery [9,13]. To our knowledge, the genome sequence of *E. coioides *is still unavailable, and this has hindered the progress of immunological and developmental research. To overcome this obstacle, the 454 pyrosequencing technology was applied to determine the transcriptome sequence of *E. coioides *spleen tissue and a comparative analysis of transcriptome data between the control and the SGIV infected group was performed in this study. The data obtained disclosed a great deal of novel gene information in marine fish and suggested that several intracellular immune signaling pathways were involved in virus infection. These results will shed new light on the understanding of marine fish defense mechanisms to viral pathogens.

## Results

### Sequence analysis of ESTs from different cDNA libraries

Sequencing data from two different libraries was submitted to the NCBI database (accession number is SRA040065.1). In the control (C) and the SGIV (V) infected spleen libraries, a total of 428867 and 446009 ESTs were sequenced, respectively. Following adaptor sequence and low quality sequences trimming 407,027 (C) and 421,141 (V), high-quality ESTs were obtained from the two libraries. After sequence assembly, 60,322 non-redundant ESTs were generated in the control library, including 36,076 singlets and 24,246 contigs with an average length of 504 bp. In the infected library, 66,063 non-redundant ESTs were generated, including 40,527 singlets and 25,536 contigs, with an average length of 547 bp (Table 1).

**Table 1:** Summary of EST data in mock- and SGIV-infected grouper spleen cDNA libraries.

Categary	spleen Library	
	
	Mock-infected	SGIV infected
Total sequenced cDNA	428867	446009
High quality ESTs	407027	421141
Total bp	10620407	11962121
Number of contigs	24246	25536
Number of singlets	36076	40527
N50 of contigs (bp)	504	547

N50 = median length of the sequences of all the contigs

**Figure 1:**
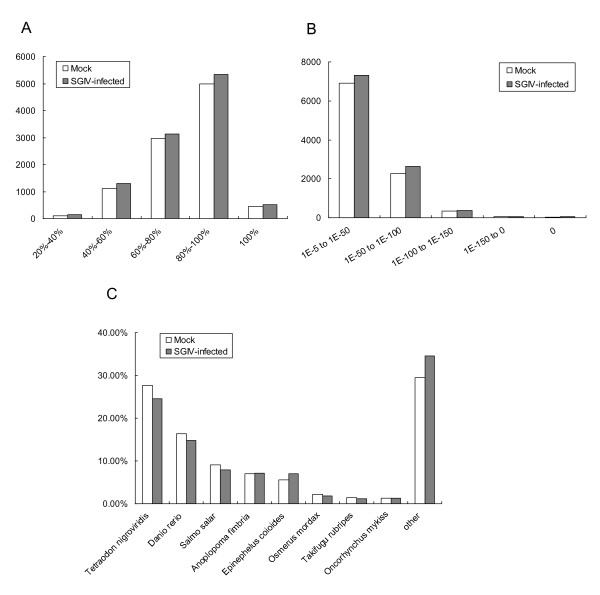
**Characteristics of homology search of ESTs against the nr database**. (A) E-value distribution of BLAST hits for each unique sequence with a cut-off E-value of 1.0E-5. (B) Similarity distribution of the top BLAST hits for each sequence. (C) Species distribution is shown as a percentage of the total homologous sequences with an E-value of at least 1.0E-5. We used the first hit of each sequence for analysis.

All the contigs and singlets were designated as unique sequences and used for further comparative sequence analysis between the two libraries. After a homology search in the non-redundant protein database at the National Center for Biotechnology Information (NCBI), a total of 9,616 (C) and 10,426 (V) unique sequences showed significant BLASTX hits of known protein sequences. The distribution of significant BLASTX hits over different organisms was analyzed. Due to the lack of *E. coioides *genomic information, the majority of sequences in the two libraries matched genes or fragments from Tetraodon nigroviridis (Figure 1).

### Functional annotation based on GO, COG and KEGG analysis

**Figure 2:**
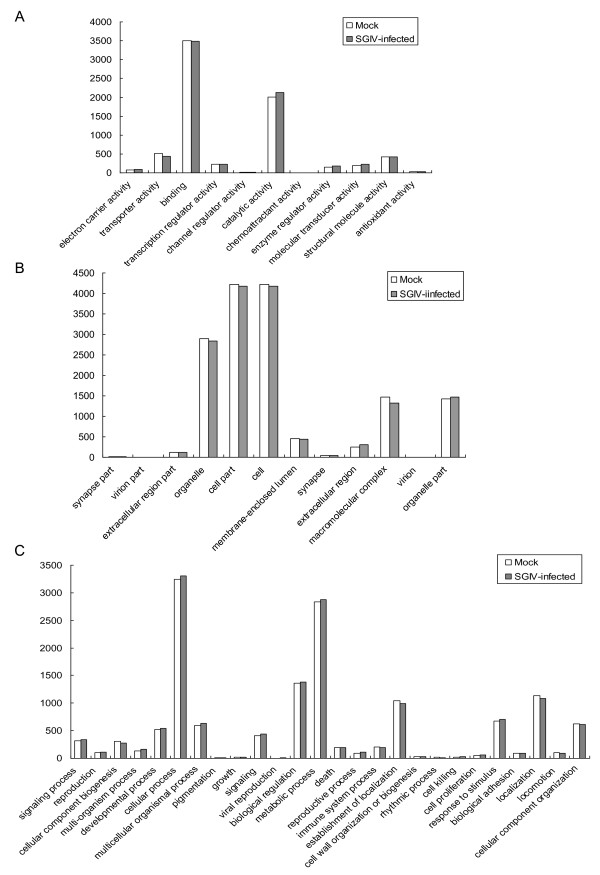
**GO annotations of non-redundant sequences in mock and SGIV infected libraries**. Most non-redundant sequences can be divided into three major categories, including molecular function (A), cellular component (B), and biological process (C).

The putative functions of unique sequences in two different libraries were analyzed according to Gene Ontology (GO) and Clusters of Orthologous Groups of protein (COGs) classifications. Analysis of GO categories showed that the functional distribution of the genes of the two libraries was similar. A total of 14,166 and 14,352 unique sequences map to biological processes, 15,130 and 14,923 sequences map to cellular components, and 7,137 and 7,252 sequences map to molecular functions in the control and SGIV infected libraries, respectively. In both libraries, most of the corresponding biological process genes were involved in cellular processes, biological regulation and metabolic processes. Most of the cellular component genes encode proteins associated with parts of cells and cell organelles; most of the molecular function genes were associated with binding, catalytic activity, and transporter activity (Figure 2).

**Figure 3:**
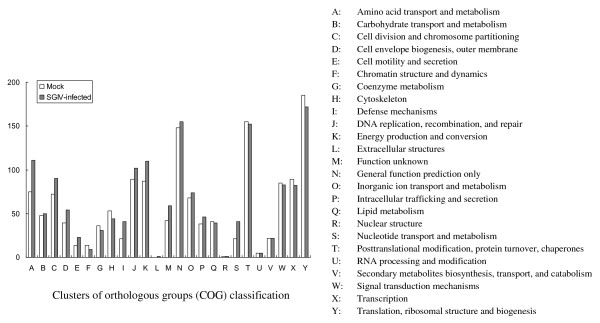
**Histogram presentation of clusters of orthologous groups (COG) classification in mock and SGIV infected libraries**.

Classification of the unigenes into COG categories is critical for functional and evolutionary studies [14]. Among the 25 COG categories, the cluster in the control library for "translation, ribosomal structure and biogenesis" represented the largest group (185 ESTs), followed by the "posttranslational modification, protein turnover, chaperones" and "general function prediction" clusters. Similarly, in the SGIV infected library, the cluster for "translation, ribosomal structure and biogenesis" represented the largest group (172 ESTs) followed by "general function prediction" and "posttranslational modification, protein turnover, chaperones" clusters (Figure 3).

KEGG is a pathway-based categorization of orthologous genes that provides useful information for predicting functional profiles of genes [15]. In this study the unique sequences of two libraries were categorized within the KEGG database. The matched sequences were involved in metabolism processes, cellular processes, signal transduction and cell cycles. Partial KEGG pathways associated with immune and inflammation responses are listed in Table 2. The conserved MAPK signaling molecules can be found in control (C) and SGIV-infected libraries (V), which contained 65 and 71 ESTs, respectively (Additional file 1). In addition, a large number of ESTs were involved in RIG-I signaling pathway (C, 21 hits; V, 20 hits), TLR signaling pathway (C, 28 hits; V, 26 hits), chemokine signaling pathway (C, 62 hits; 73 hits) and P53 signaling pathway (C, 22 hits; V, 25 hits) in two different libraries (Additional file 2 and 3). Many ESTs associated with mammalian signaling pathway genes, including MAP Kinase phosphatase 1 (MKP-1), Nur77, stimulator of interferon genes (STING), and tripartite motif protein (finTRIM) were initially disclosed in marine fish.

**Table 2:** Number of ESTs involved in KEGG pathway (number of ESTs > 10)

Pathway	Pathway Name	Number of ESTs	
		Control library	SGIV infected library
04010	MAPK signaling pathway	65	71
04062	Chemokine signaling pathway	62	73
04120	Ubiquitin mediated proteolysis	57	69
04660	T cell receptor signaling pathway	39	35
03050	Proteasome	39	37
04620	Toll-like receptor signaling pathway	28	26
04662	B cell receptor signaling pathway	28	30
04630	Jak-STAT signaling pathway	26	25
04020	Calcium signaling pathway	25	36
04210	Apoptosis	24	26
04115	p53 signaling pathway	22	25
04622	RIG-I-like receptor pathway	21	20
04350	TGF-beta signaling pathway	17	23
04150	mTOR signaling pathway	13	13

### Putative genes involved in up-regulation or down-regulation during SGIV infection

Among unique sequences that shared > 30% identity (E value < 1e-5) to known genes in the NCBI database, 2,057 genes were cross-expressed in both the control and the SGIV-infected libraries. Using the Fisher's exact test based on the number of homologous ESTs, we found that 755 genes were significantly up-regulated, while 695 genes were significantly down-regulated in response to SGIV infection. A large number of genes were only present in either the control library or the SGIV-infected library. The up-regulated and down-regulated partial genes are listed in Tables 3 and 4, respectively. The alternated genes included cytoskeletal genes, enzymes, and other immune-related genes, such as chemokines, interleukins and interferon-induced proteins. These genes have different expression patterns during SGIV infection, which implies that they may play an important role in physiological processes associated with SGIV infection.

**Table 3:** Unique genes with increased expression in spleen after SGIV infection

Gene name	species	expression in normal	Expression in infection	Fisher p_value
Profilin-2	Rattus norvegicus	305	29051	0
60S ribosomal protein	Homo sapiens	251	23569	0
Eotaxin	Mus musculus	3169	17689	0
Granulins	Rattus norvegicus	1141	15720	0
Leukocyte cell-derived chemotaxin-2	Mus musculus	4809	10637	0
Thioredoxin	Ictalurus punctatus	542	7343	0
Saposin-C	Cavia porcellus	43	5116	0
Cystatin-B 1	Macaca fuscata	92	4388	0
Perforin-1	Mus musculus	105	4097	0
Scavenger receptor cysteine-rich type 1 protein	Canis familiaris	911	3958	0
Eukaryotic translation initiation factor 4 gamma 2	Gallus gallus	573	3829	0
Gamma-glutamyl hydrolase	Homo sapiens	133	2638	0
Legumain	Bos taurus	445	2572	0
Proteasome subunit alpha type-7	Carassius auratus	35	2452	0
Glyceraldehyde 3-phosphate dehydrogenase,	Danio rerio	103	1983	0
Transcription factor BTF3	Mus musculus	83	1770	0
Src-like-adapter	Mus musculus	84	1371	2.02E-303
Keratin 8	Gallus gallus	182	1586	5.86E-283
Galectin-3-binding protein B	Danio rerio	450	2187	8.23E-277
SUMO-conjugating enzyme	Xenopus tropicalis	137	1307	8.94E-243
C-C motif chemokine 18	Macaca mulatta	282	1667	3.00E-242
Galectin-3-binding protein A	Danio rerio	371	1747	3.37E-216
Hemoglobin subunit beta-1	Pseudaphritis urvillii	241	1383	1.38E-197
Keratin 18	Oncorhynchus mykiss	1661	3701	7.37E-180
Eukaryotic translation initiation factor 3	Bos taurus	67	844	4.27E-174
Cytochrome b	Tropheus moorii	6544	9945	1.29E-163
Natural killer cell protease 1	Rattus norvegicus	227	1138	1.95E-148
T-complex protein 1	Gallus gallus	50	669	6.61E-141
RNA-binding protein 5	Xenopus tropicalis	45	605	7.97E-128
Cathepsin H	Sus scrofa	391	1347	3.79E-125
Beta-2-glycoprotein 1	Bos taurus	433	1389	7.97E-119
Dipeptidyl peptidase 1	Bos taurus	55	586	1.36E-114
Proto-oncogene vav	Mus musculus	98	693	8.51E-113
Proteasome subunit alpha type-4	Homo sapiens	684	1761	2.56E-111
Actin-related protein 2/3 complex subunit 5	Mus musculus	90	656	1.35E-108
Proteasome subunit beta type-6-B like	Salmo salar	469	1361	8.49E-103
RING-box protein 2	Mus musculus	42	497	3.76E-101
NADH-ubiquinone oxidoreductase chain 1	Carassius auratus	330	1102	2.68E-99
Phospholipid hydroperoxide glutathione peroxidase	Sus scrofa	590	1527	1.66E-97
Retinol-binding protein 1	Bos taurus	164	767	1.80E-95
NudC domain-containing protein 2	Rattus norvegicus	81	576	3.36E-94
Lysozyme g	Epinephelus coioides	164	742	5.02E-90
Voltage-gated hydrogen channel 1	Danio rerio	217	742	4.09E-69
Ubiquitin-like modifier-activating enzyme 5	Xenopus laevis	179	670	8.17E-69
LRR and PYD domains-containing protein 1	Homo sapiens	205	714	6.02E-68
Cytochrome c oxidase subunit 6B1	Tarsius syrichta	60	395	2.33E-62
Ig lambda chain	Homo sapiens	31	315	2.99E-61
Interleukin-8	Equus caballus	155	572	4.14E-58
Fibroleukin	Homo sapiens	611	1281	3.75E-56
Rho GDP-dissociation inhibitor 1	Macaca fascicularis	325	844	4.69E-55
Ependymin	Notemigonus crysoleucas	70	386	5.52E-55
Fucolectin-4	Anguilla japonica	1519	2434	1.29E-50
Complement factor H	Homo sapiens	131	459	1.88E-44
Eukaryotic initiation factor 4A-III	Xenopus tropicalis	56	282	4.32E-38
Cysteine and glycine-rich protein 2	Mus musculus	61	290	1.68E-37
Proliferating cell nuclear antigen	Haplochromis burtoni	237	575	3.64E-34
Secernin-3	Danio rerio	64	277	2.42E-33
Ubiquitin-conjugating enzyme E2	Mus musculus	74	286	4.09E-31
Heat shock cognate 71 kDa protein	Oryzias latipes	25	181	8.31E-31

**Table 4:** Unique genes with decreased expression in spleen after SGIV infection

swissprot_annotation	species	Expression in control	Expression in infection	Fisher p_value
H-2 class II histocompatibility antigen	Mus musculus	11365	1044	0
Glutathione peroxidase 1	Bos taurus	3469	509	0
Endothelial differentiation-related factor 1	Xenopus laevis	5830	172	0
Inositol-3-phosphate synthase 1-A	Xenopus laevis	1618	148	0
Palmitoyl-protein thioesterase 1	Mus musculus	6570	92	0
Proteasome subunit beta type-2	Bos taurus	2244	84	0
Cytochrome c oxidase subunit 1	Gadus morhua	7690	66	0
Myosin light polypeptide 6	Rattus norvegicus	1733	65	0
Eukaryotic translation initiation factor 3 subunit	Danio rerio	1765	60	0
Gamma-interferon-inducible lysosomal thiol reductase	Homo sapiens	2962	55	0
Mid1-interacting protein 1-like	Danio rerio	3667	54	0
Elongation factor 1-gamma	Carassius auratus	1821	51	0
Plastin-2	Danio rerio	1777	51	0
ribosomal protein S8	Rattus norvegicus	1737	48	0
Stefin-C	Bos taurus	1991	46	0
Complement C1q subcomponent subunit A	Bos taurus	1511	43	0
ribosomal protein L22	Xenopus tropicalis	2286	33	0
ribosomal protein L18	Oreochromis niloticus	1598	29	0
Nucleolar protein 16	Tetraodon nigroviridis	1949	377	9.53E-251
Transcription initiation factor TFIID	Pongo abelii	2123	469	6.58E-246
Transforming protein RhoA	Rattus norvegicus	1258	202	4.46E-184
Peroxiredoxin	Cynops pyrrhogaster	2563	982	7.87E-157
Myosin regulatory light chain 2	Gallus gallus	5634	3124	2.64E-154
Heat shock 70 kDa protein	Canis familiaris	612	31	7.54E-140
Ras-related C3 botulinum toxin substrate 2	Bos taurus	928	167	1.56E-126
SH3 domain-containing protein	Mus musculus	609	48	1.33E-123
Rab GDP dissociation inhibitor beta	Rattus norvegicus	602	48	9.70E-122
Cystatin-A5	Sus scrofa	2339	994	4.94E-120
Radixin	Mus musculus	675	77	8.53E-119
Interferon-inducible GTPase 1	Homo sapiens	805	181	4.16E-93
C-X-C chemokine receptor type 4	Papio anubis	1929	851	1.51E-92
AIG2-like domain-containing protein	Danio rerio	617	128	1.59E-76
Major complex class I-related gene	Mus musculus	1058	366	1.83E-76
Thioredoxin-dependent peroxide reductase,	Homo sapiens	769	209	1.20E-74
Death-associated protein-like 1-B	Xenopus laevis	1417	638	9.05E-66
RING finger protein 10	Mus musculus	277	24	3.52E-55
Mannan-binding lectin serine protease 2	Mus musculus	2177	1249	6.20E-55
GTP-binding nuclear protein	Salmo salar	3669	2432	9.56E-54
Regulator of G-protein signaling 8	Danio rerio	1206	639	6.61E-39
Matrix metalloproteinase-9	Homo sapiens	199	21	2.33E-37
Bleomycin hydrolase	Gallus gallus	418	125	2.50E-37
Src kinase-associated phosphoprotein 2	Takifugu rubripes	257	47	8.27E-36
Interferon-induced protein 44-like	Mus musculus	352	94	1.44E-35
Protein disulfide-isomerase A4	Rattus norvegicus	191	21	2.56E-35
Macrophage mannose receptor 1	Homo sapiens	257	49	8.14E-35
Prothymosin alpha-B	Danio rerio	186	21	4.75E-34
Ubiquitin-conjugating enzyme E2	Drosophila melanogaster	344	97	6.36E-33
Myosin-8	Canis familiaris	1023	547	1.60E-32
cAMP-responsive element-binding protein	Danio rerio	684	312	8.58E-32
Programmed cell death protein 10	Mus musculus	383	123	1.42E-31
Peroxisomal membrane protein 2	Bos taurus	612	267	3.46E-31
E3 ubiquitin-protein ligase	Homo sapiens	220	41	2.02E-30
Myoferlin	Xenopus tropicalis	262	62	4.71E-30
Ras-related protein Rab7	Gossypium hirsutum	223	50	6.44E-27
Dynactin subunit 6	Homo sapiens	242	61	2.40E-26
protease regulatory subunit 4	Rattus norvegicus	229	55	4.43E-26
Interferon regulatory factor 1	Gallus gallus	446	183	1.06E-25
Hephaestin-like protein 1	Mus musculus	276	88	2.05E-23
Galectin-8	Homo sapiens	158	29	1.39E-22
Apoptosis-associated speck-like protein	Danio rerio	497	233	2.41E-22

### Validation of the changes in gene expression by quantitative real-time PCR

**Figure 4:**
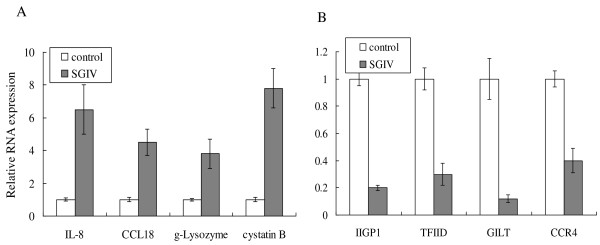
**The differential expression of selected genes was validated by qRT-PCR**. Relative expression of genes with increased abundance (A) or decreased abundance (B) was detected. The relative gene expression in grouper injected with PBS (control) was defined as 1, and that in SGIV infected grouper (48 h p.i.) was indicated by the fold increase or decrease compared to the control.

To validate whether the up-regulated or down-regulated genes identified by statistical analysis were involved in SGIV infection, we detected the relative expression of partial genes using quantitative real time-PCR (qRT-PCR). As shown in Figure 4, the relative expression of IL-8, Chemokine (C-C motif) ligand 18 (CCL18), g-type lysozyme (g-lysozyme) and cystatin B increased significantly after SGIV infection, compared with the expression of these genes in the control fish. In contrast, the expression of the interferon-inducible GTPase 1 (IIGP1), transcription Factor II D (TFIID), gamma interferon (IFN-γ)-inducible lysosomal thiol reductase (GILT) and C-C chemokine receptor type 4 (CCR4) decreased after SGIV infection. Thus, these results suggested that SGIV infection modulated numerous host gene expressions for the completion its life cycle.

## Discussion

An increasing number of reports reveal that transcriptome sequencing of cDNA has became an efficient strategy for generating enormous sequences that represent expressed genes [16]. Transcriptomes from a number of species including those from *Drosophila melanogaster*, yeast, *Caenorhabditis elegans *and various mammals and plants were carried out for different purposes [17-21]. However, genome and transcriptome data for many "lower" vertebrate species, particularly marine fishes, have not been disclosed. To our knowledge, a limited numbers of *E. coioides *genes were cloned and characterized, based on the bioinformatic analysis, including those involved in immune responses after pathogenic attack, growth and development [22-27]. Given that the spleen is one of the most important organs associated with immune responses in fish and is also the main target organ for SGIV infection, the transcriptome sequencing of the *E. coioides *spleen can be expected to provide a significant number of ESTs for marine fish immune responses and contribute to understanding iridovirus-host interactions [5].

After removal of overlapping sequences between the control and SGIV-infected libraries, we obtained 65374 non-redundant consensus sequences from *E. coioides*. With the exception of sequences related to cellular structure and metabolism, abundant sequences were found to be homologous to known immune-relevant genes in other species, based on the BLAST, Conserved Domain Database (CDD), and SWISS-PROT annotation [28-30]. More than 80 sequences shared homology to signaling molecules of the mammalian mitogen-activated protein kinase (MAPK) pathways, such as critical molecules associated with extracellular signal-regulated kinase (ERK), p38 MAPK, Ras, RSK2, MKK4, MKK7, ASK1, MEK1/2 and Raf1. The mammalian MAPK signaling pathway was activated during virus infection and contributed to virus replication [31-33]. Although the MAPK signaling molecules including ERK, c-Jun N-terminal kinase (JNK) and p38 MAPK were activated in the spleens of SGIV-infected fish (EAGS) cells, identifying the exact roles of these molecules during SGIV replication will benefit from the *E. coioides *EST information [34,35]. With the exception of homologue components in the MAPK cascade, different members of interferon-related genes were obtained, including the interferon-induced protein viperin, the interferon-stimulated gene 15 (ISG15), interferon-induced protein 35 kD (IFP35), interferon-stimulated gene 56 (ISG56), and interferon regulatory factors (IRF-1, IRF-2, IRF-3, IRF-4, IRF-5, IRF-7, IRF-8 and IRF-9). Interferon-induced, or stimulated, genes were important for the resistance of the host to virus infection, including virus entry, replication and release [36-38]. The *E. coioides *IRF-1, IRF-2 and IRF-7 genes have been cloned and characterized and IRF-7 was confirmed as being vitally important for SGIV replication [39,40]. Human ISG15 expression is strongly up-regulated during viral infections, such as human cytomegalovirus (HCMV) and herpes simplex virus (HSV), and ISG15 up-regulation was considered to be involved in different strategies relating to the antiviral response [41-44]. IFP35 and ISG56 were also involved in the cellular antiviral response against virus infection [38,45]. A detailed investigation on the functions of *E. coioides *interferon-related genes during SGIV infection will contribute greatly to understanding how the SGIV exploited, or evaded, the host interferon immune response.

We also obtained sequences that shared homology to SGIV-encoded immune evasion genes, including lipopolysaccharide-induced tumor necrosis factor-α factor (LITAF), tumor necrosis factor receptor (TNFR), ubiquitin and Bcl-2 [46-48]. Iridovirus-encoded LITAF and Bcl-2 could mediate the fate of host cells by regulating apoptosis [47,48]. It has been reported that many viral immune evasion genes are considered as "stolen" mimics from the host and such viruses may interfere with the host response by modulating or disrupting the function of corresponding host genes [49-51]. The discovery of these sequences will be helpful in studies on host-virus interactions. In addition, we also found that other molecules such as lectin, hepcidin, lysozyme and antimicrobial peptide are involved in immune responses. The functions of these genes during virus infection will be investigated in the further studies.

Based on results from exploratory statistical analysis, we identified genes that are up-regulated or down-regulated after SGIV infection. The present data from qRT-PCR analysis validated the hypothesis that expression of partial genes is regulated by SGIV infection, including cytokine, cytokine receptor and transcription factor, apoptosis-associated genes, interferon-related genes, and cytoskeleton genes. Previous studies indicated that the expression of different groups of genes relating to cellular structure, apoptosis, gene transcription and immune regulation were altered in response to virus infections or other stimuli [37,52-54]. Further research into the roles of these differentially-expressed genes will contribute to an increased understanding of the critical events that take place during SGIV replication.

## Conclusions

In summary, we studied the immune response of marine fish to virus infection using SGIV infected *E. coioides *as a model. More than 400 000 high-quality ESTs were obtained from the *E. coioides *spleen cDNA library by 454 sequencing. These unique sequences contribute greatly to the investigation into changes in gene expression patterns and their molecular functions during pathogens infection, and also provide an abundant data source for the identification of novel genes in *E. coioides*. This gene information can be used to provide further insights into the functions of chemokines, proinflammatory factors, interferon-induced genes and other cytokines and will thus stimulate further study on the immune response of *E. coioides *to pathogens. The experimental validation of the gene expression alterations during SGIV infection provides new insights into understanding iridovirus-host interactions.

## Methods

### *E. coioides *and virus challenge

To construct spleen cDNA libraries, groupers (*E. coioides) *of 15 cm total length were obtained from a local farm in Guangzhou, China. Sampling detection indicated that these fish tested negative to SGIV infection. All the fish were maintained in a laboratory recirculating seawater system at 25-30°C for 2 weeks. Healthy fish that displayed normal levels of activity were used in this study. The virus suspension used as a challenge was collected from SGIV-infected GS cells. The fish were challenged by injecting with 0.2 ml of the SGIV suspension (1 × 10^5 ^TCID_50_/ml). As a control, an equal volume of PBS was likewise injected. At 48 h post-infection, fish were sacrificed and tissue samples were taken from the spleens. These were stored in liquid nitrogen for later RNA extraction.

### RNA extraction, cDNA library construction and 454 sequencing

Total RNA was extracted from the spleens of the control and SGIV-infected fish using an SV total RNA Isolation kit (Promega). The cDNA library preparation and 454-pyrosequencing were performed as described in Salem et al. [11]. This encompassed a number of procedures as described below. In brief, the first and second strand cDNA were synthesized from 1 μg of total RNA using the SMART PCR cDNA Synthesis Kit (Clonetech, USA) with modified 3' primer 5'-AAGCAGTGGTATCAACGCAGAGTGCAG(T20)VN-3' that contained a BsgI cleavage site. Then the double-stranded cDNA was digested with BsgI for 16 h and cleaned with a QIAquick Minelute PCR purification column (Qiagen, CA). The purified cDNA was sheared into fragments ranging from about 400 to 1000 base pairs by nebulization. After the short fragments (< 400 bp) were removed by AMPure bead (Agencourt), samples were processed with GS FLX Titanium General DNA Library Preparation Kit (Roche) following the manufacturer's instructions. Sequencing was carried out using Roche 454 Genome Sequencer FLX instrument. All the obtained data were submitted to NCBI database.

### Data analysis

To analyze the data generated by the FLX sequencer, the sequences of adapters, low complexity and low-quality sequences were filtered out by using Seq-clean and LUCY software [55]. The screened high-quality sequences were *de novo *assembled used CAP3 software under default parameters [56]. ESTs that did not form contigs were designated as singlets. Putative functions of all the unique sequences (contigs and singlets) were predicted using local BLASTall programs against sequences in the NCBI non-redundant (nr) protein database and the swissprot database (E-value < 1e-5). Each unique sequence was used to determine the COG term, GO term, and the involvement of KEGG pathway database [14,15].

To compare the gene expression profile between two different libraries, EST occurrence was evaluated statistically. The abundance of unique sequence, expressed as an increase or decrease if the number of hits in SGIV-infected library, was classed as "significantly more" or "significantly less" than that of a normal library. The statistical significance of ESTs with different abundance values was determined using Fisher's exact test [57,58]. A P value of < 0.05 was considered as statistically significant.

### Quantitative real-time PCR

Quantitative real-time PCR was carried out using a LightCycler ^® ^480 Real-Time PCR System (Roche), with SYBR Green as the fluorescent dye, according to the manufacturer's protocol (TOYOBO). Different genes including cytokines (IL-8, CCL18), cytokine receptors (CCR4), transcription factors (TFIID), apoptosis-associated genes (cystatin B), interferon-related genes (GILT, IIGP1) and others (lysozyme G) were used for validation. Primer sequences are listed in Table 5. Reaction conditions were as follows: 95°C for 1 min, followed by 40 cycles at 94°C for 15 s and at 60°C for 1 min; all the reactions were performed in biological triplicates and samples were normalized using β-actin. Results were expressed as relative fold of β-actin in each experiment, as mean ± SD.

**Table 5:** Primers used in this study.

Name	Sequence of primers (5'→3')
Cystatin-PF	GTGATGAGGTAAAGCCCAGTGCGGAG
Cystatin-PR	TGGCAGTGGTTTGAAAACACGGAGGT
CCL18-PF	TGCTTTCCTCAGTGATCTGCCAG
CCL18-PR	AGATGCGACGACCCTTTTTTGAA
CCR4-PF	ACAACAGCCAAGCCACAGGAAGC
CCR4-PR	CAGGTGAAAAAACAAACAATGAA
IL8-PF	GTGTCAACCCAGTGCTGTATGCCTT
IL8-PR	TTCAAAGTGTCTCTCTGGTCGTCTC
IIGP1-PF	ACCACCTTAGAGGCTACACCATACCCC
IIGP1-PR	TCTCCTGAGCGAGTTTCACATCATTTT
GILT-PF	TGTTCCTAACTGAGATGCTCTTCCCC
GILT-PR	ATGTTGCCCTGACATTCTGGTGGTC
g-lysozyme-PF	CCTATAATACCTACGGGCTGATG
g-lysozyme-PR	TAGGCTGCTATCCCACCTTTCA
TFIID-PF	CCAGGAGGATGAGGAGGAGGAG
TFIID-PR	GCTGTATGGAGGAGAAAGGGTT

## Abbreviations

SGIV: Singapore grouper iridovirus; GO: Gene ontology; COG: clusters of orthologous group; KEGG: Kyoto Encyclopedia of Genes and Genomes; MAPK: mitogen-activated protein kinase; OSGIV: orange-spotted grouper iridovirus; TGIV: Taiwan grouper iridovirus; TRIM: tripartite motif protein; STING: stimulator of interferon genes; IIGP1: interferon-inducible GTPase 1; TFIID: transcription Factor II D; GILT: gamma interferon (IFN-γ)-inducible lysosomal thiol reductase; CCR4: C-C chemokine receptor type 4; LITAF: lipopolysaccharide-induced tumor necrosis factor-α factor; TNFR: tumor necrosis factor receptor.

## Authors' contributions

YHH and XHH performed the bioinformatics analysis, qRT-PCR validation and drafted the manuscript. YY, HCC, JC, ZLOY and PRW participated in culturing *E. coioides*, RNA extraction, primer design, and bioinformatics analysis. YHH and QQW contributed to the experimental design and manuscript editing. All authors read and approved the final manuscript.

## Supplementary Material

Additional file 1 **Figure S1. The ESTs involved in MAPK signaling pathway in KEGG database**. (A), ESTs in control library hit to MAPK signaling pathway in KEGG data base. (B), ESTs in infected library hit to MAPK signaling pathway in KEGG database. Click here for file

Additional file 2 **Figure S2. ESTs in control library hit to the RIG-I (A), TLR (B), chemokine (C), P53 signaling pathway (D)**. Click here for file

Additional file 3 **Figure S3. ESTs in SGIV infected library hit to the RIG-I (A), TLR (B), chemokine (C), P53 signaling pathway (D)**. Click here for file

## References

[B1] Zhou L Gui JF Molecular mechanisms underlying sex change in hermaphroditic groupers Fish Physiol Biochem 2010 36 181 193 10.1007/s10695-008-9219-0 20467860

[B2] Marino G Azzurro E Massari A Finoia MG Mandich A Reproduction in the dusky grouper from the southern Mediterranean J Fish Biol 2001 58 908 927

[B3] Walker PJ Winton JR Emerging viral diseases of fish and shrimp Vet Res 2010 41 51 10.1051/vetres/2010022 20409453 PMC2878170

[B4] Whittington RJ Becker JA Dennis MM Iridovirus infections in finfish - critical review with emphasis on ranaviruses J Fish Dis 2010 33 95 122 10.1111/j.1365-2761.2009.01110.x 20050967

[B5] Qin QW Shi C Gin KY Lam TJ Antigenic characterization of a marine fish iridovirus from grouper, Epinephelus spp J Virol Methods 2002 106 89 96 10.1016/S0166-0934(02)00139-8 12367733

[B6] Chao CB Chen CY Lai YY Lin CS Huang HT Histological, ultrastructural, and in situ hybridization study on enlarged cells in grouper Epinephelus hybrids infected by grouper iridovirus in Taiwan (TGIV) Dis Aquat Organ 2004 58 127 142 15109134 10.3354/dao058127

[B7] Kai YH Su HM Tai KT Chi SC Vaccination of grouper broodfish (Epinephelus tukula) reduces the risk of vertical transmission by nervous necrosis virus Vaccine 2010 28 996 1001 10.1016/j.vaccine.2009.10.132 19954763

[B8] Lü L Zhou SY Chen C Weng SP Chan SM He JG Complete genome sequence analysis of an iridovirus isolated from the orange-spotted grouper, Epinephelus coioides Virology 2005 339 81 100 10.1016/j.virol.2005.05.021 15964605

[B9] Vera JC Wheat CW Fescemyer HW Frilander MJ Crawford DL Hanski I Marden JH Rapid transcriptome characterization for a nonmodel organism using 454 pyrosequencing Mol Ecol 2008 17 1636 1647 10.1111/j.1365-294X.2008.03666.x 18266620

[B10] Alagna F D'Agostino N Torchia L Servili M Rao R Pietrella M Giuliano G Chiusano ML Baldoni L Perrotta G Comparative 454 pyrosequencing of transcripts from two olive genotypes during fruit development BMC Genomics 2009 10 399 10.1186/1471-2164-10-399 19709400 PMC2748093

[B11] Salem M Rexroad CE Wang J Thorgaard GH Yao J Characterization of the rainbow trout transcriptome using Sanger and 454-pyrosequencing approaches BMC Genomics 2010 11 564 10.1186/1471-2164-11-564 20942956 PMC3091713

[B12] Guo S Zheng Y Joung JG Liu S Zhang Z Crasta OR Sobral BW Xu Y Huang S Fei Z Transcriptome sequencing and comparative analysis of cucumber flowers with different sex types BMC Genomics 2010 11 384 10.1186/1471-2164-11-384 20565788 PMC2897810

[B13] Emrich SJ Barbazuk WB Li L Schnable PS Gene discovery and annotation using LCM-454 transcriptome sequencing Genome Res 2007 17 69 73 17095711 10.1101/gr.5145806 PMC1716268

[B14] Tatusov RL Galperin MY Natale DA Koonin EV The COG database: a tool for genome-scale analysis of protein functions and evolution Nucleic Acids Res 2000 28 33 36 10.1093/nar/28.1.33 10592175 PMC102395

[B15] Kanehisa M Goto S KEGG: kyoto encyclopedia of genes and genomes Nucleic Acids Res 2000 28 27 30 10.1093/nar/28.1.27 10592173 PMC102409

[B16] Morozova O Hirst M Marra MA Applications of new sequencing technologies for transcriptome analysis Annu Rev Genomics Hum Genet 2009 10 135 151 10.1146/annurev-genom-082908-145957 19715439

[B17] Graveley BR Brooks AN Carlson JW Duff MO Landolin JM Yang L Artieri CG van Baren MJ Boley N Booth BW Brown JB Cherbas L Davis CA Dobin A Li R Lin W Malone JH Mattiuzzo NR Miller D Sturgill D Tuch BB Zaleski C Zhang D Blanchette M Dudoit S Eads B Green RE Hammonds A Jiang L Kapranov P Langton L Perrimon N Sandler JE Wan KH Willingham A Zhang Y Zou Y Andrews J Bickel PJ Brenner SE Brent MR Cherbas P Gingeras TR Hoskins RA Kaufman TC Oliver B Celniker SE The developmental transcriptome of Drosophila melanogaster Nature 2011 471 473 479 10.1038/nature09715 21179090 PMC3075879

[B18] Wilhelm BT Marguerat S Watt S Schubert F Wood V Goodhead I Penkett CJ Rogers J Bähler J Dynamic repertoire of a eukaryotic transcriptome surveyed at single-nucleotide resolution Nature 2008 453 1239 1243 10.1038/nature07002 18488015

[B19] Mizrachi E Hefer CA Ranik M Joubert F Myburg AA De novo assembled expressed gene catalog of a fast-growing Eucalyptus tree produced by Illumina mRNA-Seq BMC Genomics 2010 11 681 10.1186/1471-2164-11-681 21122097 PMC3053591

[B20] Fehniger TA Wylie T Germino E Leong JW Magrini VJ Koul S Keppel CR Schneider SE Koboldt DC Sullivan RP Heinz ME Crosby SD Nagarajan R Ramsingh G Link DC Ley TJ Mardis ER Next-generation sequencing identifies the natural killer cell microRNA transcriptome Genome Res 2010 20 1590 1604 10.1101/gr.107995.110 20935160 PMC2963822

[B21] Li P Ponnala L Gandotra N Wang L Si Y Tausta SL Kebrom TH Provart N Patel R Myers CR Reidel EJ Turgeon R Liu P Sun Q Nelson T Brutnell TP The developmental dynamics of the maize leaf transcriptome Nat Genet 2010 42 1060 1067 10.1038/ng.703 21037569

[B22] Jin JY Zhou L Wang Y Li Z Zhao JG Zhang QY Gui JF Antibacterial and Antiviral Roles of a Fish β-Defensin Expressed Both in Pituitary and Testis PLoS One 2010 5 e12883 10.1371/journal.pone.0012883 21188147 PMC3004800

[B23] Zhou JG Wei JG Xu D Cui HC Yan Y Ou-Yang ZL Huang XH Huang YH Qin QW Molecular cloning and characterization of two novel hepcidins from orange-spotted grouper, Epinephelus coioides Fish Shellfish Immunol 2011 30 559 568 10.1016/j.fsi.2010.11.021 21145974

[B24] Cui H Yan Y Wei J Hou Z Huang Y Huang X Qin Q Cloning, characterization, and expression analysis of orange-spotted grouper (Epinephelus coioides) ILF2 gene (EcILF2) Fish Shellfish Immunol 2011 30 378 388 10.1016/j.fsi.2010.11.015 21109006

[B25] Dong H Zeng L Duan D Zhang H Wang Y Li W Lin H Growth hormone and two forms of insulin-like growth factors I in the giant grouper (Epinephelus lanceolatus): molecular cloning and characterization of tissue distribution Fish Physiol Biochem 2010 36 201 212 10.1007/s10695-008-9231-4 20467861

[B26] Shi Y Zhang Y Li S Liu Q Lu D Liu M Meng Z Cheng CH Liu X Lin H Molecular identification of the Kiss2/Kiss1ra system and its potential function during 17alpha-methyltestosterone-induced sex reversal in the orange-spotted grouper, Epinephelus coioides Biol Reprod 2010 83 63 74 10.1095/biolreprod.109.080044 20375257

[B27] Xia W Zhou L Yao B Li CJ Gui JF Differential and spermatogenic cell-specific expression of DMRT1 during sex reversal in protogynous hermaphroditic groupers Mol Cell Endocrinol 2007 263 156 172 10.1016/j.mce.2006.09.014 17092636

[B28] Junker V Contrino S Fleischmann W Hermjakob H Lang F Magrane M Martin MJ Mitaritonna N O'Donovan C Apweiler R The role SWISS-PROT and TrEMBL play in the genome research environment J Biotechnol 2000 78 221 234 10.1016/S0168-1656(00)00198-X 10751683

[B29] Ye J McGinnis S Madden TL BLAST: improvements for better sequence analysis Nucleic Acids Res 2006 34 W6 9 10.1093/nar/gkl164 16845079 PMC1538791

[B30] Marchler-Bauer A Anderson JB Chitsaz F Derbyshire MK DeWeese-Scott C Fong JH Geer LY Geer RC Gonzales NR Gwadz M He S Hurwitz DI Jackson JD Ke Z Lanczycki CJ Liebert CA Liu C Lu F Lu S Marchler GH Mullokandov M Song JS Tasneem A Thanki N Yamashita RA Zhang D Zhang N Bryant SH CDD: specific functional annotation with the Conserved Domain Database Nucleic Acids Res 2009 37 D205 210 10.1093/nar/gkn845 18984618 PMC2686570

[B31] Xing Z Cardona CJ Anunciacion J Adams S Dao N Roles of the ERK MAPK in the regulation of proinflammatory and apoptotic responses in chicken macrophages infected with H9N2 avian influenza virus J Gen Virol 2010 91 343 351 10.1099/vir.0.015578-0 19864500

[B32] Regan AD Cohen RD Whittaker GR Activation of p38 MAPK by feline infectious peritonitis virus regulates pro-inflammatory cytokine production in primary blood-derived feline mononuclear cells Virology 2009 384 135 143 10.1016/j.virol.2008.11.006 19058829 PMC7103373

[B33] Holloway G Coulson BS Rotavirus activates JNK and p38 signaling pathways in intestinal cells, leading to AP-1-driven transcriptional responses and enhanced virus replication J Virol 2006 80 10624 10633 10.1128/JVI.00390-06 16928761 PMC1641755

[B34] Huang XH Huang YH Ouyang ZL Xu LX Yan Y Cui HC Han X Qin QW Singapore grouper iridovirus, a large DNA virus, induces nonapoptotic cell death by a cell type dependent fashion and evokes ERK signaling Apoptosis 2011 16 831 845 10.1007/s10495-011-0616-y 21656148

[B35] Huang XH Huang YH Ouyang ZL Cai J Yan Y Qin QW Roles of Stress-Activated Protein Kinases in the replication of Singapore grouper iridovirus and regulation of the inflammatory responses in grouper cells J Gen Virol 92 1292 1301 10.1099/vir.0.029173-0 21402598

[B36] Jiang D Weidner JM Qing M Pan XB Guo H Xu C Zhang X Birk A Chang J Shi PY Block TM Guo JT Identification of five interferon-induced cellular proteins that inhibit west nile virus and dengue virus infections J Virol 2010 84 8332 8341 10.1128/JVI.02199-09 20534863 PMC2916517

[B37] Wang X Hinson ER Cresswell P The interferon-inducible protein viperin inhibits influenza virus release by perturbing lipid rafts Cell Host Microbe 2007 2 96 105 10.1016/j.chom.2007.06.009 18005724

[B38] Li Y Li C Xue P Zhong B Mao AP Ran Y Chen H Wang YY Yang F Shu HB ISG56 is a negative-feedback regulator of virus-triggered signaling and cellular antiviral response Proc Natl Acad Sci USA 2009 106 7945 7950 10.1073/pnas.0900818106 19416887 PMC2683125

[B39] Shi Y Zhu XP Yin JK Zhang QY Gui JF Identification and characterization of interferon regulatory factor-1 from orange-spotted grouper (Epinephelus coioides) Mol Biol Rep 2010 37 1483 1493 10.1007/s11033-009-9544-0 19444647

[B40] Cui H Yan Y Wei J Huang X Huang Y Ouyang Z Qin Q Identification and functional characterization of an interferon regulatory factor 7-like (IRF7-like) gene from orange-spotted grouper, Epinephelus coioides Dev Comp Immunol 2011 35 672 684 10.1016/j.dci.2011.01.021 21295068

[B41] Lenschow DJ Lai C Frias-Staheli N Giannakopoulos NV Lutz A Wolff T Osiak A Levine B Schmidt RE García-Sastre A Leib DA Pekosz A Knobeloch KP Horak I Virgin HW IFN-stimulated gene 15 functions as a critical antiviral molecule against influenza, herpes, and Sindbis viruses Proc Natl Acad Sci USA 2007 104 1371 1376 10.1073/pnas.0607038104 17227866 PMC1783119

[B42] Okumura A Lu G Pitha-Rowe I Pitha PM Innate antiviral response targets HIV-1 release by the induction of ubiquitin-like protein ISG15 Proc Natl Acad Sci USA 2006 103 1440 1445 10.1073/pnas.0510518103 16434471 PMC1360585

[B43] Nicholl MJ Robinson LH Preston CM Activation of cellular interferon-responsive genes after infection of human cells with herpes simplex virus type 1 J Gen Virol 2000 81 2215 2218 10950979 10.1099/0022-1317-81-9-2215

[B44] Broering R Zhang X Kottilil S Trippler M Jiang M Lu M Gerken G Schlaak JF The interferon stimulated gene 15 functions as a proviral factor for the hepatitis C virus and as a regulator of the IFN response Gut 2010 59 1111 1119 10.1136/gut.2009.195545 20639253

[B45] Tan J Qiao W Wang J Xu F Li Y Zhou J Chen Q Geng Y IFP35 is involved in the antiviral function of interferon by association with the viral tas transactivator of bovine foamy virus J Virol 2008 82 4275 4283 10.1128/JVI.02249-07 18305040 PMC2293045

[B46] Song WJ Qin QW Qiu J Huang CH Wang F Hew CL Functional genomics analysis of Singapore grouper iridovirus: complete sequence determination and proteomic analysis J Virol 2004 78 12576 12590 10.1128/JVI.78.22.12576-12590.2004 15507645 PMC525058

[B47] Lin PW Huang YJ John JA Chang YN Yuan CH Chen WY Yeh CH Shen ST Lin FP Tsui WH Chang CY Iridovirus Bcl-2 protein inhibits apoptosis in the early stage of viral infection Apoptosis 2008 13 165 176 10.1007/s10495-007-0152-y 17955372

[B48] Huang XH Huang YH Gong J Yan Y Qin QW Identification and characterization of a putative lipopolysaccharide-induced TNF-alpha factor (LITAF) homolog from Singapore grouper iridovirus Biochem Biophys Res Commun 2008 373 140 145 10.1016/j.bbrc.2008.06.003 18554501

[B49] Viswanathan K Früh K DeFilippis V Viral hijacking of the host ubiquitin system to evade interferon responses Curr Opin Microbiol 2010 13 517 523 10.1016/j.mib.2010.05.012 20699190 PMC2939720

[B50] Alcami A Viral mimicry of cytokines, chemokines and their receptors Nat Rev Immunol 2003 3 36 50 10.1038/nri980 12511874

[B51] Ploegh HL Viral strategies of immune evasion Science 1998 280 248 253 10.1126/science.280.5361.248 9535648

[B52] Yeh CH Chen YS Wu MS Chen CW Yuan CH Pan KW Chang YN Chuang NN Chang CY Differential display of grouper iridovirus-infected grouper cells by immunostaining Biochem Biophys Res Commun 2008 372 674 680 10.1016/j.bbrc.2008.05.126 18519026

[B53] Chen LM Tran BN Lin Q Lim TK Wang F Hew CL iTRAQ analysis of Singapore grouper iridovirus infection in a grouper embryonic cell line J Gen Virol 2008 89 2869 2876 10.1099/vir.0.2008/003681-0 18931085

[B54] Xu D Wei J Cui H Gong J Yan Y Lai R Qin Q Differential profiles of gene expression in grouper Epinephelus coioides, infected with Singapore grouper iridovirus, revealed by suppression subtractive hybridization and DNA microarray J Fish Biol 2010 77 341 360 10.1111/j.1095-8649.2010.02676.x 20646160

[B55] Chou HH Holmes MH DNA sequence quality trimming and vector removal Bioinformatics 2001 17 1093 1104 10.1093/bioinformatics/17.12.1093 11751217

[B56] Huang X Madan A CAP3:A DNA sequence assembly program Genome Res 1999 9 868 877 10.1101/gr.9.9.868 10508846 PMC310812

[B57] Leu JH Chang CC Wu JL Hsu CW Hirono I Aoki T Juan HF Lo CF Kou GH Huang HC Comparative analysis of differentially expressed genes in normal and white spot syndrome virus infected Penaeus monodon BMC Genomics 2007 8 120 10.1186/1471-2164-8-120 17506900 PMC1888707

[B58] Zhang Z Wang Y Wang S Liu J Warren W Mitreva M Walter RB Transcriptome analysis of female and male Xiphophorus maculatus Jp 163 A PLoS One 2011 6 e18379 10.1371/journal.pone.0018379 21483681 PMC3071723

