# Supplementary material for: Transcriptome analysis of orange-spotted grouper (Epinephelus coioides) spleen in response to Singapore grouper iridovirus
Source: BMC Genomics. 2011 Nov 12;12:556. doi: 10.1186/1471-2164-12-556 (PMC3226587; doi:10.1186/1471-2164-12-556)
Supplement: Additional file 1 — Figure S1. The ESTs involved in MAPK signaling pathway in KEGG database. (A), ESTs in control library hit to MAPK signaling pathway in KEGG data base. (B), ESTs in infected library hit to MAPK signaling pathway in KEGG database. [file 1471-2164-12-556-S1.DOC]

Additional file 1

Figure S1. The ESTs involved in MAPK signaling pathway in KEGG database. (A), ESTs in control library hit to MAPK signaling pathway in KEGG data base. (B), ESTs in infected library hit to MAPK signaling pathway in KEGG database.


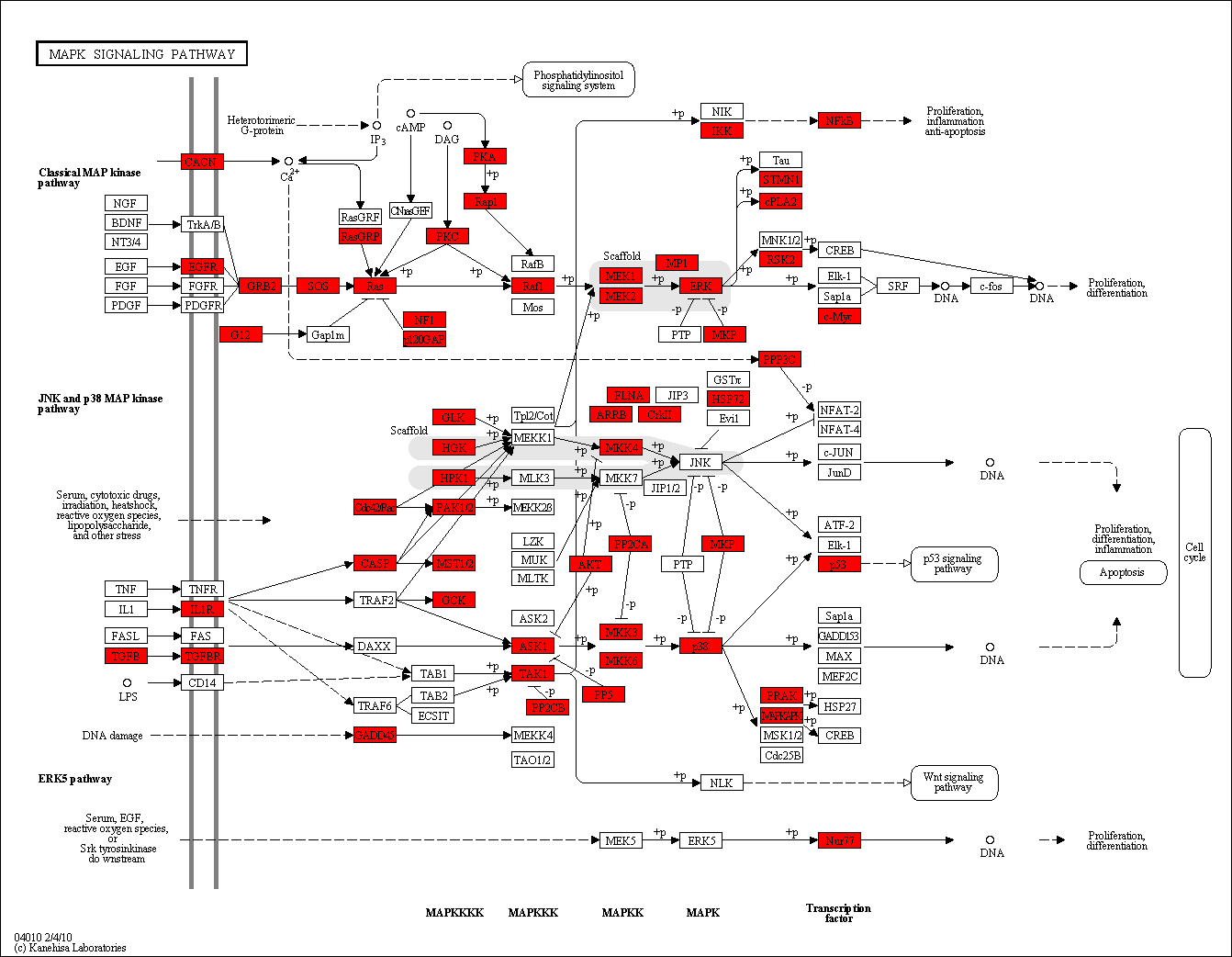


A


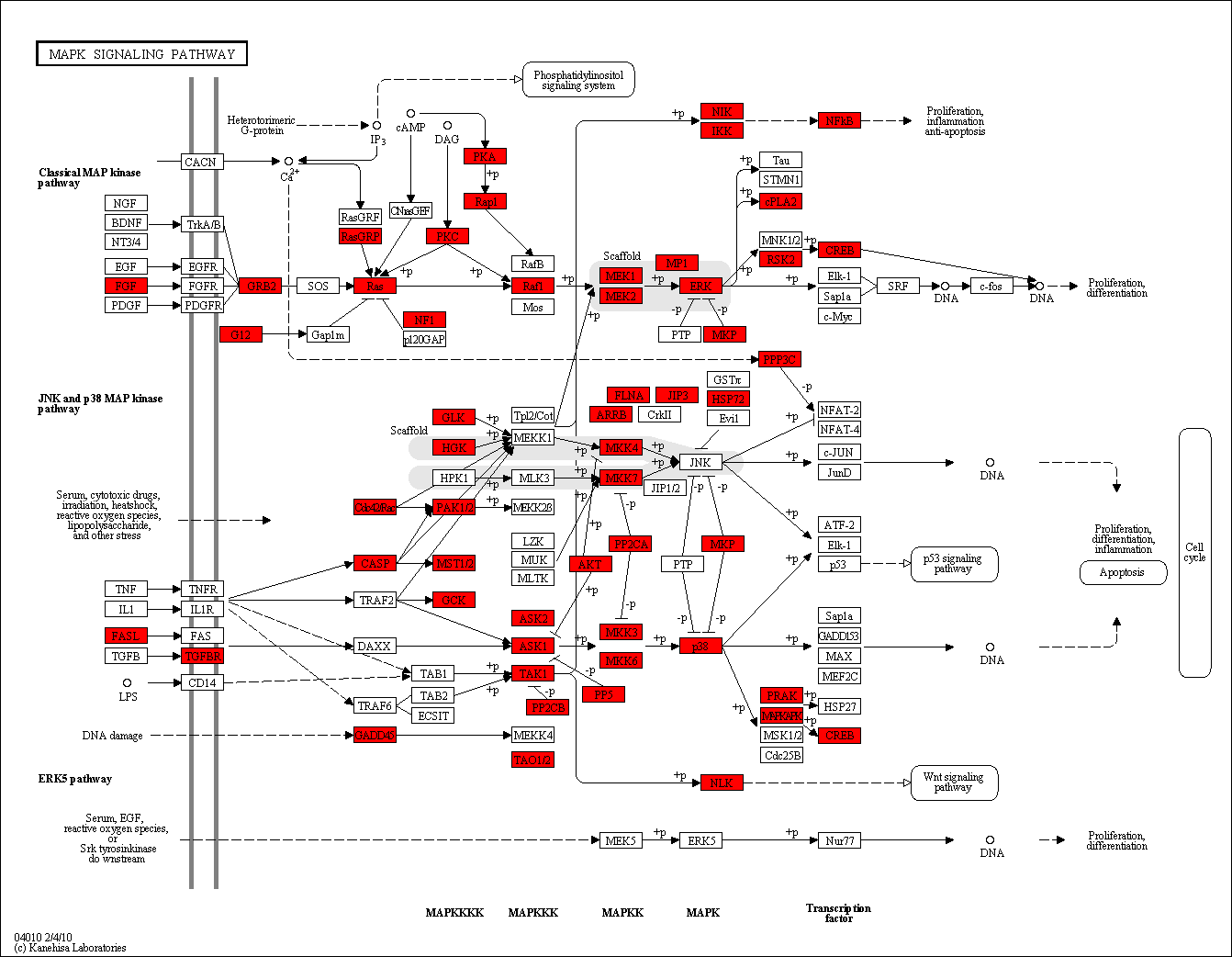


B

Figure S1
