# Supplementary material for: Transcriptome analysis of orange-spotted grouper (Epinephelus coioides) spleen in response to Singapore grouper iridovirus
Source: BMC Genomics. 2011 Nov 12;12:556. doi: 10.1186/1471-2164-12-556 (PMC3226587; doi:10.1186/1471-2164-12-556)
Supplement: Additional file 2 — Figure S2. ESTs in control library hit to the RIG-I (A), TLR (B), chemokine (C), P53 signaling pathway (D). [file 1471-2164-12-556-S2.DOC]

Additional file 2

Figure S2. ESTs in control library hit to the RIG-I (A), TLR (B), chemokine (C), P53 signaling pathway (D).

A


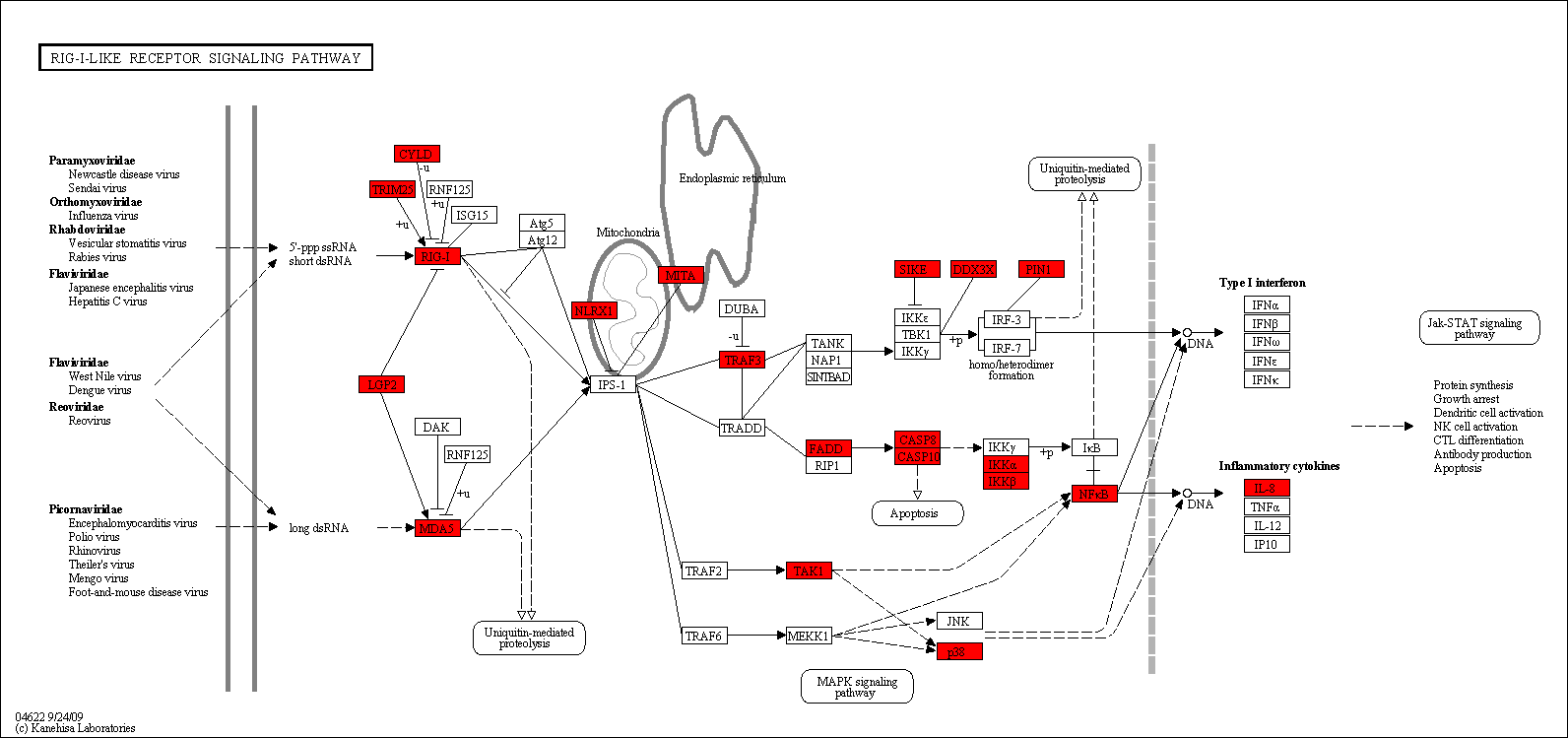

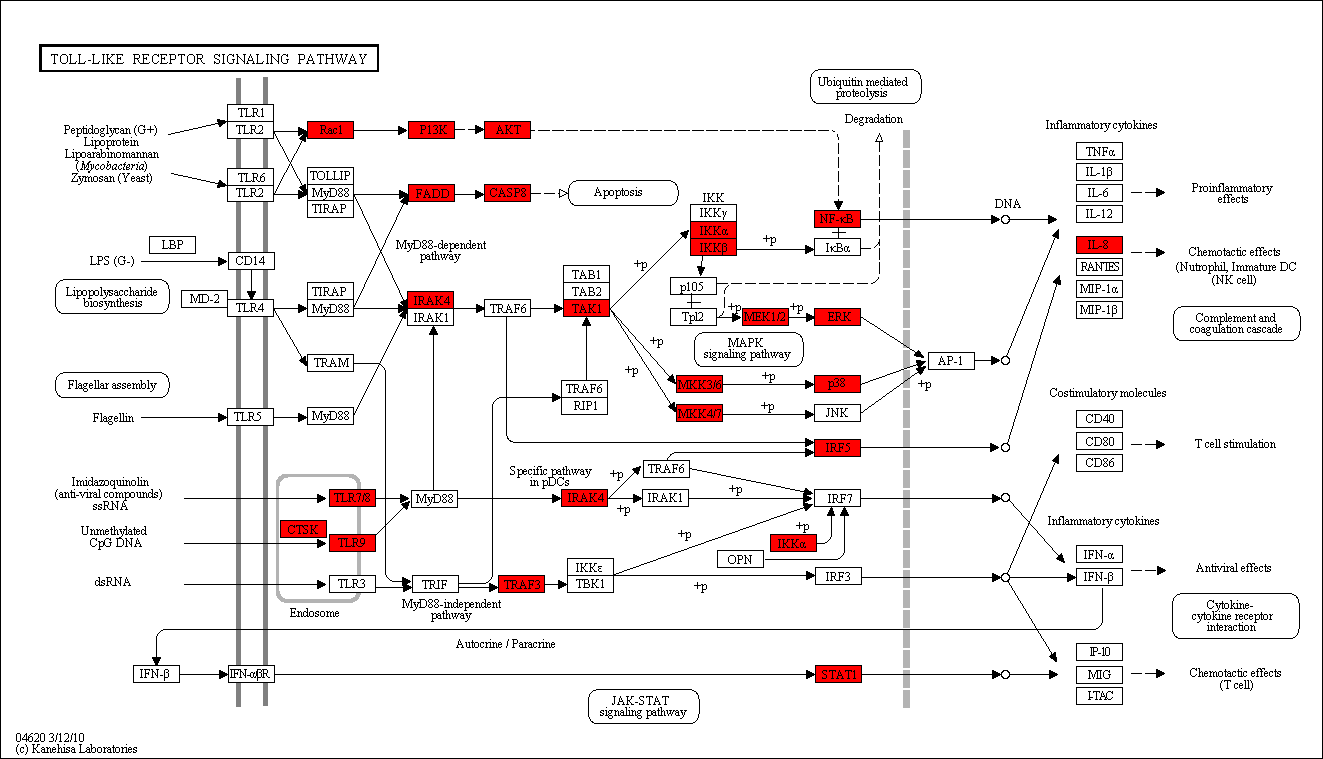


B


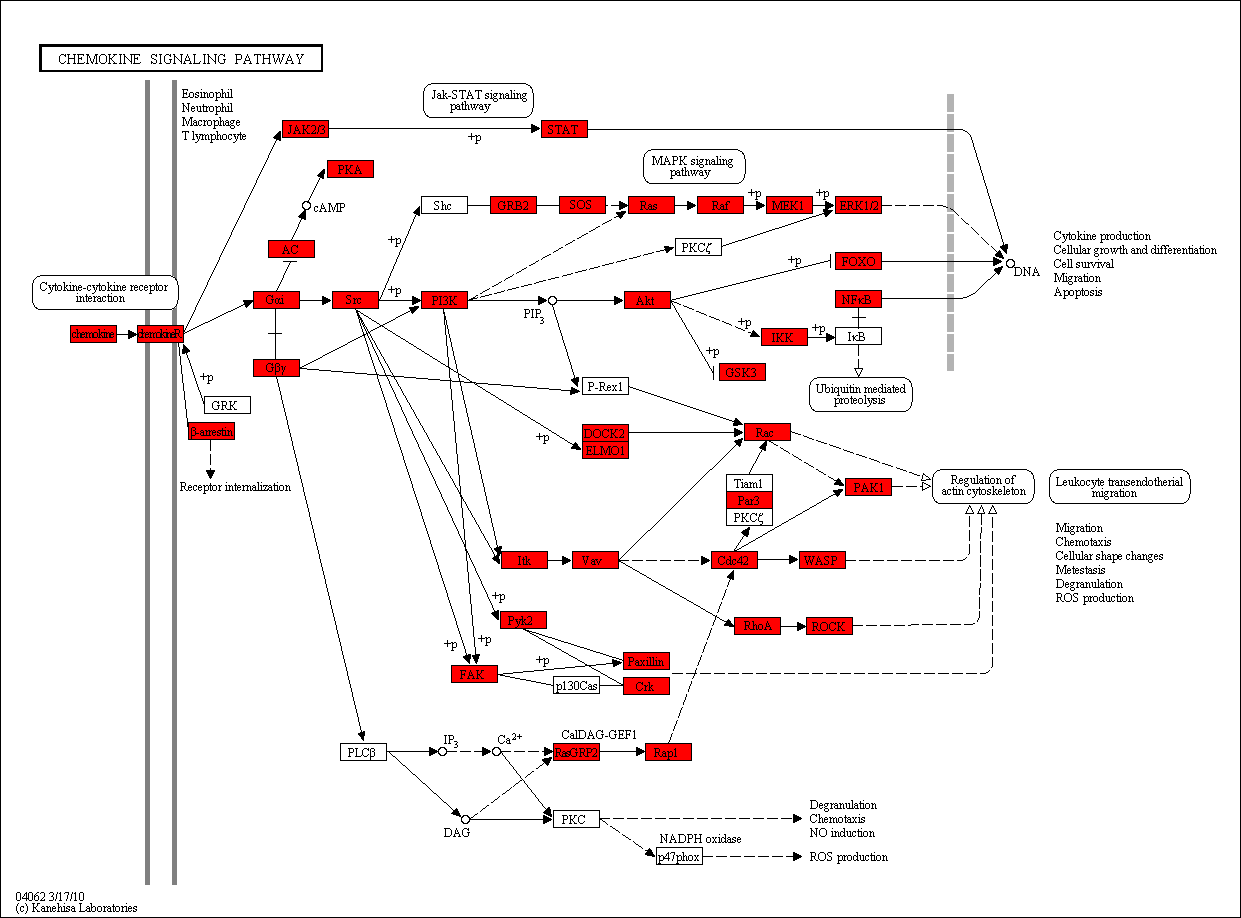

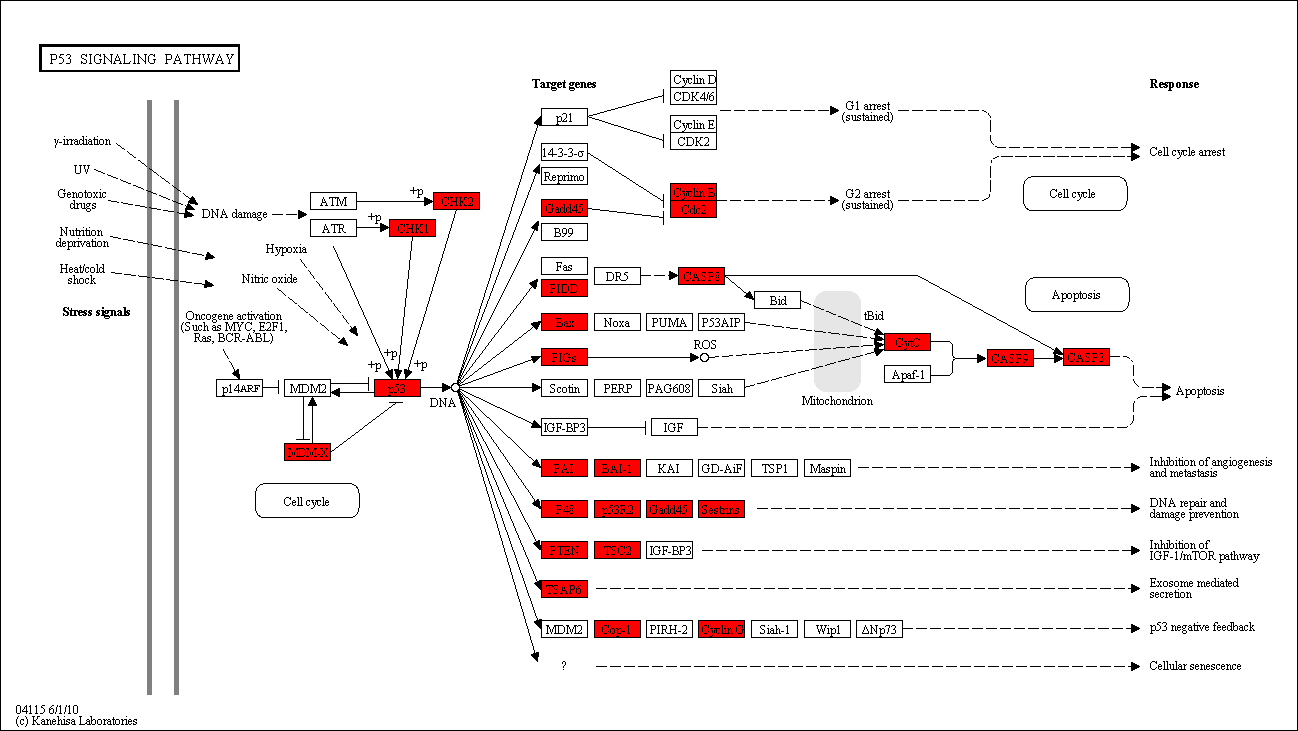


C

D

Figure S2
