# Supplementary material for: Transcriptome analysis of orange-spotted grouper (Epinephelus coioides) spleen in response to Singapore grouper iridovirus
Source: BMC Genomics. 2011 Nov 12;12:556. doi: 10.1186/1471-2164-12-556 (PMC3226587; doi:10.1186/1471-2164-12-556)
Supplement: Additional file 3 — Figure S3. ESTs in SGIV infected library hit to the RIG-I (A), TLR (B), chemokine (C), P53 signaling pathway (D). [file 1471-2164-12-556-S3.DOC]

Additional file 3

Figure S3. ESTs in SGIV infected library hit to the RIG-I (A), TLR (B), chemokine (C), P53 signaling pathway (D).


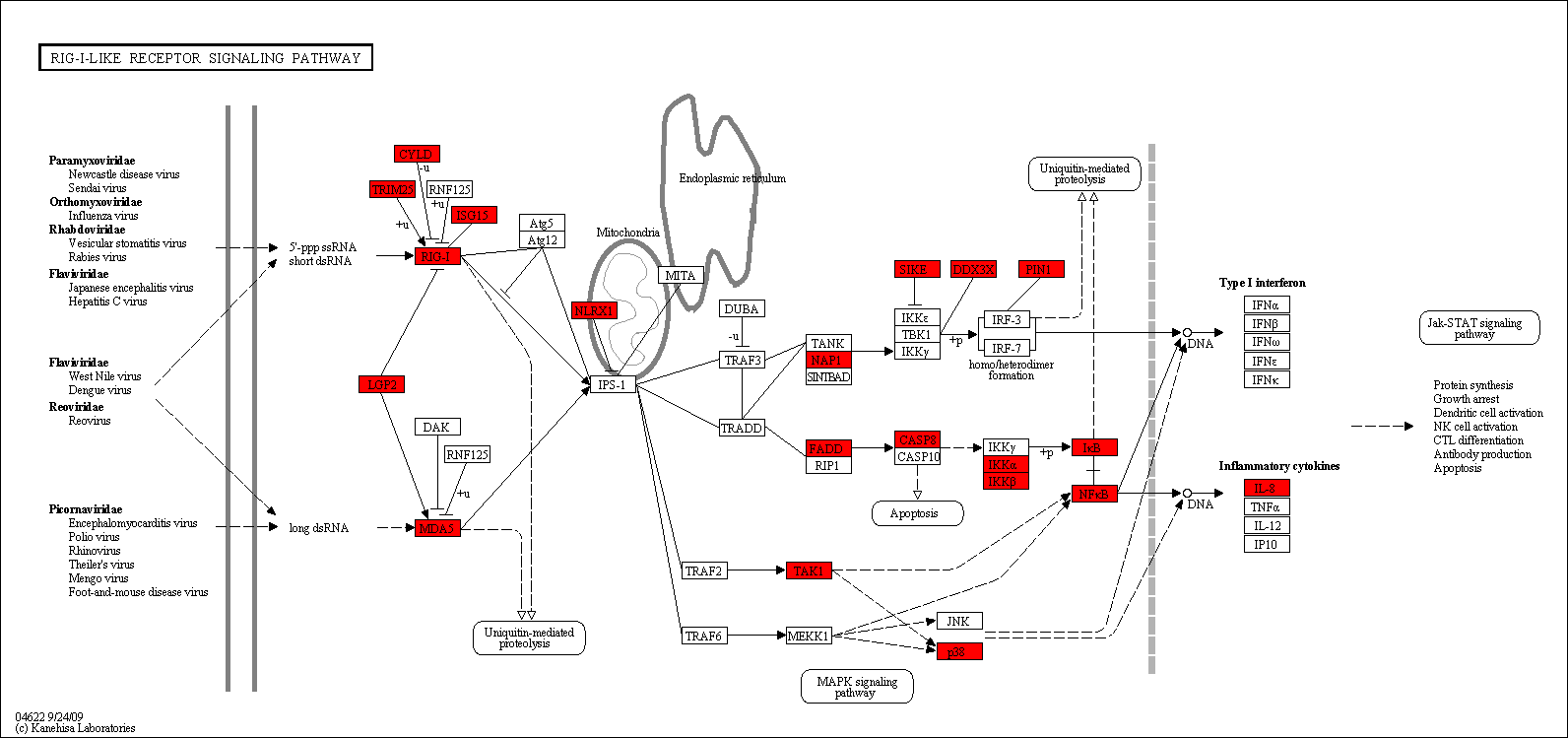


A

B


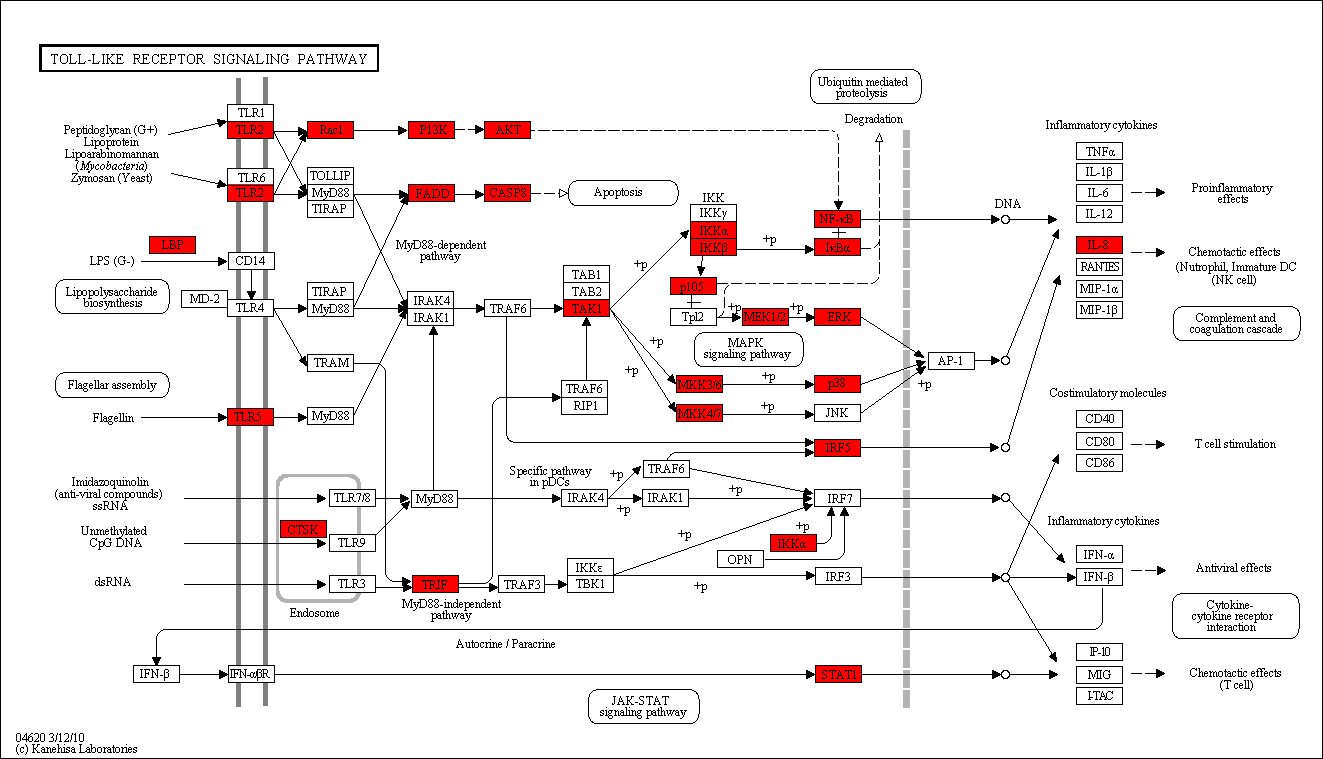


C


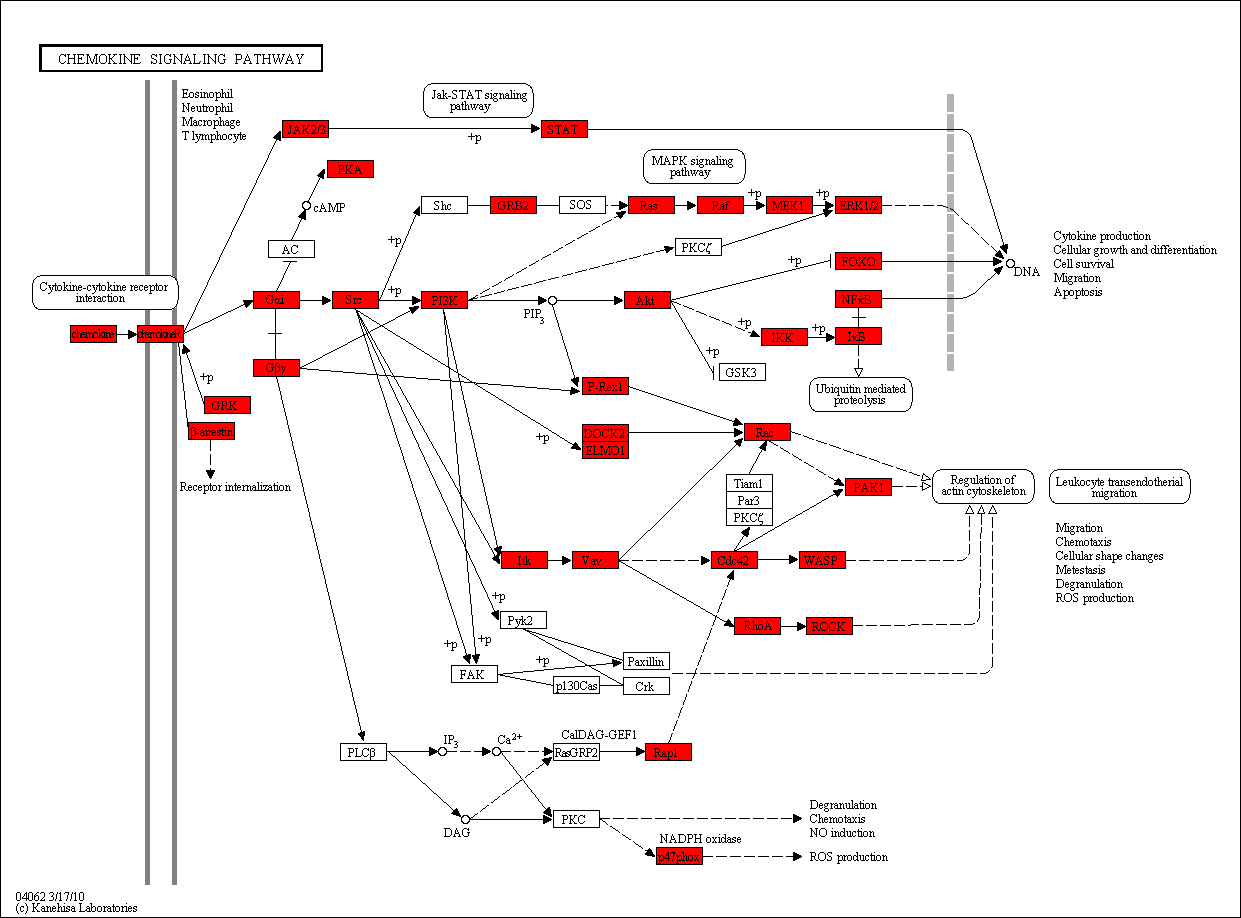


D


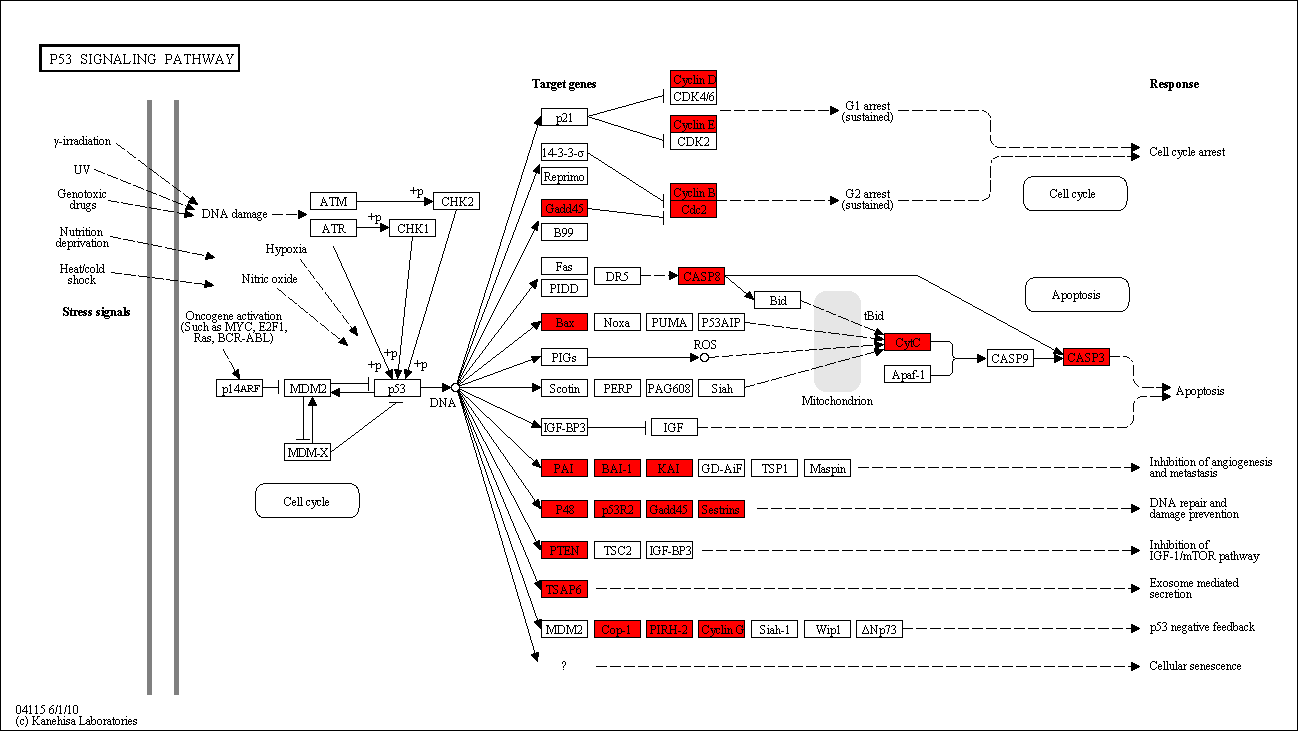


Figure S3
